# Risk Factors and Prevalence of *mcr-1*-Positive *Escherichia coli* in Fecal Carriages Among Community Children in Southern Taiwan

**DOI:** 10.3389/fmicb.2021.748525

**Published:** 2021-11-19

**Authors:** Pin-Chieh Wu, Ming-Fang Cheng, Wan-Ling Chen, Wan-Yu Hung, Jiun-Ling Wang, Chih-Hsin Hung

**Affiliations:** ^1^Institute of Biotechnology and Chemical Engineering, I-Shou University, Kaohsiung, Taiwan; ^2^Department of Physical Examination Center, Kaohsiung Veterans General Hospital, Kaohsiung, Taiwan; ^3^Department of Nursing, Meiho University, Pingtung, Taiwan; ^4^Department of Pediatrics, Kaohsiung Veterans General Hospital, Kaohsiung, Taiwan; ^5^Department of Medicine, National Yang-Ming University, Taipei, Taiwan; ^6^Department of Nursing, Fooyin University, Kaohsiung, Taiwan; ^7^Department of Internal Medicine, National Cheng Kung University Hospital, Tainan, Taiwan; ^8^Department of Medicine, College of Medicine, National Cheng Kung University, Tainan, Taiwan

**Keywords:** *mcr-1*, risk factor, prevalence, community children, fecal carriage, Taiwan

## Abstract

Colistin is the last resort antimicrobial for treating multidrug-resistant gram-negative bacterial infections. The plasmid-mediated colistin resistance gene, *mcr-1*, crucially influences colistin’s resistance transmission. Human fecal carriages of *mcr-1*-positive *Escherichia coli (E. coli)* were detected in many regions worldwide; however, only a few studies have focused on children. Therefore, we identified the prevalence and risk factors of *mcr-1*-positive *E. coli* in fecal carriages among community children in Southern Taiwan. In this study, 510 stool samples were collected from April 2016 to August 2019 from the pediatric department at a medical center in Southern Taiwan. These samples were collected within 3 days after admission and were all screened for the presence of the *mcr-1* gene. Diet habits, travel history, pet contact, and medical history were also obtained from participants to analyze the risk factors of their fecal carriages to *mcr-1*-positive *E. coli*. Antimicrobial susceptibility testing was determined using the VITEK 2 system and the broth microdilution test. Twelve *mcr-1*-positive *E. coli.* were isolated from 2.4% of the fecal samples. Through multivariate analysis, frequent chicken consumption (at least 3 times per week) had a significantly positive association with the presence of *mcr-1*-positive *E. coli* in fecal carriages (adjust odds ratio 6.60, 95% confidence interval1.58– 27.62, *p* = 0.033). Additionally, multidrug resistance was more common in *mcr-1*-positive *E. coli.* (75.0% vs. 39.5%, *p* = 0.031) than in non-*mcr-1*-positive *Escherichia coli*. Furthermore, the percentage of extraintestinal pathogenic *E. coli* in *mcr-1*-positive isolates was 83.3%. Some multi-locus sequence types in our *mcr-1*-positive *E. coli* were also similar to those isolated from food animals in the literature. The prevalence of fecal carriages of *mcr-1*-positive *E. coli* was low among community children in Southern Taiwan. Our data shows that chicken consumption with a higher frequency increases the risk of *mcr-1*-positive *E. coli.* in fecal carriages.

## Introduction

Antibiotic resistance is a global public health challenge of our time. Colistin (polymyxin E), first discovered in 1947, is a polycationic peptide antibiotic (Storm et al., 1977). It mainly targets the bacterial cell membrane and increases its permeability, which results in the leakage of cell contents, thereby ultimately causing cell death (Luo et al., 2020). Furthermore, it has broad-spectrum antimicrobial activities, and it is used to treat multidrug-resistant gram-negative infections (Poirel et al., 2017). Before 2015, studies showed that colistin resistance was regulated by mutation in chromosomal genes (Olaitan et al., 2014). However, in 2016, Liu et al. (2016) discovered the plasmid-mediated colistin resistance gene—*mcr-1*. Since then, the *mcr-1* gene has been identified as part of several bacterial species in humans, animals, and the environment, thus, posing a great threat to treatment (Elbediwi et al., 2019).

The human gut is a reservoir of antimicrobial resistance genes. In the gut, antimicrobial resistance genes can spread through horizontal gene transfer (Rolain, 2013; McInnes et al., 2020), which plays an important role in transmitting drug-resistant bacteria. Previous studies have also reported that the prevalence of *mcr-1-*positive *Enterobacteriaceae* in fecal carriages was 0.4–15.0% in Asia (Chan et al., 2018; Shen et al., 2018; La et al., 2019; Wu et al., 2019), 0–0.35% in Europe (Terveer et al., 2017; Zurfluh et al., 2017b), and 38.3% in South America (Giani et al., 2018), showing great variability in different geographic areas and populations. In the aspects of risk factors, several literatures from China reported antibiotic use before admission, the consumption of meat and aquaculture products were linked to *mcr-1-*positive *Enterobacteriaceae* in fecal carriages (Wang et al., 2017b; Shen et al., 2018). Furthermore, traveling to Southeast Asia has also been associated with *mcr-1*-positive *Escherichia coli* (*E. coli*) in fecal carriages (Nakayama et al., 2018). Despite these results, most studies on the risk factors for *mcr-1 Enterobacteriaceae* in fecal carriages were from the adult population, and studies among children were scarce. Furthermore, *E. coli* is a common inhabitant of the human intestinal tract and has the highest *mcr* prevalence among all bacterial species (Elbediwi et al., 2019). Therefore, we conducted a prospective study to identify the prevalence and risk factors of *mcr-1-*positive *E. coli* in fecal carriages of community children in Southern Taiwan.

## Materials and Methods

### Study Population and Data Collection

This prospective study was conducted from April 2016 to August 2019 at the pediatric department of Kaohsiung Veterans General Hospital in Southern Taiwan. Children aged below 18 years who were admitted at the pediatric department were enrolled. We contacted them as soon as they were available after admission. If they agreed to participate in the study after an interview, we described how their stool samples would be collected, after which they were given a standardized questionnaire that included demographic data, hospitalization in the last 3 months, antibiotic use in the last 3 months, history of traveling abroad, dietary habits, intake of unboiled drinking water, and history of pet contact. All participants and their legal guardians were then provided with an informed written consent. Exclusion criteria were non-willing participants, those unwilling to complete the informed written consent, those without fecal samples within 3 days after admission, and those with incomplete data in their questionnaire. The Ethics Committee of the Kaohsiung Veterans General Hospital (VGHKS 16-CT2–04, VGHKS 18-CT3–11) approved this study.

### Isolation of *E. coli* and Detection of the *mcr-1* Gene

Fecal samples were collected using cotton swabs, and then the swabs were transported in a COPAN Transystem^®^ (Copan Diagnostics, Inc., Brescia, Italy). Subsequently, the swabs were plated on a CHROMagar™ ECC plate (CHROMagar, Paris, France) and incubated without CO_2_ at 35–37^°^C for 24 h. *E. coli* colonies presented blue coloration, and up to two *E. coli* colonies were randomly selected for further analysis. Furthermore, all selected *E. coli* strains were screened for *mcr-1* genes. We used polymerase chain reaction with the primers; CLR5-F (5′-CGGTCAGTCCGTTTGTTC-3′) and CLR5-R (5′-CTTGGTCGGTCTGTA GGG-3′) for *mcr-1* gene screening (Liu et al., 2016). Additionally, all selected *E. coli* strains were spread on a CHROMagar™ ESBL plate (CHROMagar, Paris, France) to recognize the extended-spectrum β-lactamase (ESBL) *E. coli* isolates. We also checked for ESBL genes and carbapenemase genes, including; *bla_*CTX*–*M*_*, *bla_*SHV*_, bla_*TEM*_, bla_*OXA*–1_, bla_*IMP*_*, and *bla*_*VIM*_ using polymerase chain reaction (PCR) with previously described primers and methods (Chia et al., 2005; Sidjabat et al., 2009; Wu et al., 2019). Moreover, we used polymerase chain reaction to identify whether *mcr-1*-positive *E. coli* isolates belonged to extraintestinal pathogenic *E. coli* (ExPEC). Therefore, ExPEC was defined as *E. coli* isolates harboring at least two of the following five genes: S and F1C fimbriae (*focG* + *sfaS*), *kpsM II, papA, afa*, and *iutA* (Johnson et al., 2003). The *E. coli* multilocus sequence typing (MLST) scheme was also determined among *mcr-1*-positive *E. coli* isolates using seven housekeeping gene sequences (*adk, fumC, gyrB, icd, mdh, purA*, and *recA*) (Chia et al., 2005). Then, we used the goeBURST algorithm^1^ to evaluate the genetic relatedness of *mcr-1*-positive *E. coli* (Francisco et al., 2009).

### Antimicrobial Susceptibility Testing

The VITEK 2 system was used for the antimicrobial susceptibility testing of all selected *E. coli* strains. The following 17 antibiotics were included: ampicillin-sulbactam, piperacillin-tazobactam, piperacillin, cefazolin, cefoxitin, cefixime, ceftriaxone, ceftazidime, cefepime, ertapenem, imipenem, amikacin, gentamycin, ciprofloxacin, minocycline, tigecycline, and sulfamethoxazole-trimethoprim. Additionally, we used the broth dilution method to determine the antimicrobial susceptibility of colistin, sulfamethoxazole, trimethoprim, ciprofloxacin, tetracycline, meropenem, azithromycin, nalidixic acid, cefotaxime, chloramphenicol, tigecycline, ceftazidime, ampicillin, and gentamicin among all *mcr-1-*positive *E. coli* isolates. Minimum inhibitory concentration (MIC) was interpreted based on the 2020 CLSI guidelines (Clinical and Laboratory Standards Institute, 2020).

### Statistical Analysis

We used SPSS version 20.0 for Windows (SPSS Inc., Armonk, NY, United States) to perform our statistical analyses. For univariate analysis, Chi-square or Fisher’s exact tests were used to analyze categorical variables. An independent *t*-test was also used to analyze continuous variables. Furthermore, variables with a *p-*value of <0.1 in the univariate analysis were assessed using binary logistic regression analysis in the multivariate analysis. A *p*-value of<0.05 was considered statistically significant.

## Results

We collected 510 non-duplicated fecal specimens from patients in our pediatric ward. Among these specimens, 268 yielded 514 *E. coli* isolates. The characteristics of these 510 participants are demonstrated in Table 1. The participants’ ages ranged from 1 day to 17 years (mean age was 1.5 years), and males accounted for 58.6% (299/510). Hospitalization and antibiotic therapy in the last 3 months were recorded in 9.6% (49/510) and 7.8% (40/510) of participants, respectively. In addition, 7.5% (38/510) of the participants had traveled abroad in the last 12 months. Among them, 10 had been to China, 23 to Japan, 2 to Korea, 5 to Southeast Asian nations, and one to the United States. The prevalence of fecal carriages of *mcr-1*-positive *E. coli* was 2.4% (12/510). Looking further, we divided the study period into three segments. The prevalence was 1.6% (2/124) from April 2016 to March 2017, 2.5% (8/324) from April 2017 to March 2018, and 3.2% (2/62) from April 2018 to August 2019.

**TABLE 1:** Characteristics of 510 participants with and without *mcr-1 Escherichia coli* in their fecal carriages.

	*mcr-1* (+) *E. coli* (*n* = 12)	*mcr-1* (−) *E. coli* (*n* = 498)	Unadjusted OR (95% CI)	*p*-value	Adjusted OR (95% CI)	*p-*value
Age (days)	723 ± 1067	534 ± 996		0.518		
Sex (male)	11 (91.7%)	288 (57.8%)	8.02 (1.03–62.61)	0.018*		
Travel abroad in the past 12 months				0.413		
- China and Southeast Asia	1 (8.3%)	14 (2.8%)	2.99 (0.36–24.82)			
- Other countries except China	0	23(4.6%)	0.0			
- Without travel abroad	11 (91.7%)	461 (92.6%)	1.0			
Diet habit						
Pork intake				0.483		
- <1 time/week	6 (50.0%)	332 (66.7%)	1.0			
- ≥1 times/week and <3 times/week	3 (25.0%)	82 (16.5%)	2.02 (0.50–8.27)			
- ≥3 times/week	3 (25.0%)	84 (16.9%)	1.98 (0.48–8.07)			
Chicken intake				0.010*		0.033*
- <1 time/week	4 (33.3%)	341 (68.5%)	1.0		1.0	
- ≥1 times/week and <3 times/week	4 (33.3%)	109 (21.9%)	3.13 (0.77–12.72)		3.19 (0.78–13.05)	
- ≥3 times/week	4 (33.3%)	48 (9.6%)	7.10 (1.72–29.35)		6.60 (1.58–27.62)	
Duck intake				0.776		
- <1 time/week	11 (91.7%)	472 (94.8%)	1.0			
- ≥1 times/week and <3 times/week	1 (8.3%)	22 (4.4%)	1.95 (0.24–15.79)			
- ≥3 times/week	0	4 (0.8%)	0			
Beef intake				0.144		
- <1 time/week	8 (66.7%)	431 (86.5%)	1.0			
- ≥1 times/week and <3 times/week	3 (25.0%)	51 (10.2%)	3.17 (0.82–12.33)			
- ≥3 times/week	1 (8.3%)	16 (3.2%)	3.37 (0.40–28.56)			
Fish intake				0.106		
- <1 time/week	5 (41.7%)	324 (65.1%)	1.0			
- ≥1 times/week and<3 times/week	2 (16.7%)	84 (16.9%)	1.54 (0.29–80.9)			
- ≥3 times/week	5 (41.7%)	90 (18.1%)	3.60 (1.02–12.71)			
Egg intake^a^				0.285		
- <1 time/week	6 (50.0%)	329 (66.6%)	1.0			
- ≥1 times/week and <3 times/week	1 (8.3%)	55 (11.1%)	1.00 (0.12–8.44)			
- ≥3 times/week	5 (41.7%)	110 (22.3%)	2.49 (0.75–8.33)			
Unboiled water use	3 (25.0%)	128 (25.7%)	0.96 (0.26–3.61)	1.000		
Pet contact	3 (25.0%)	148 (29.7%)	0.79 (0.21–2.95)	1.000		
Medical history						
Antibiotic use in the past 3 months	3 (25.0%)	37 (7.4%)	4.15 (1.08–16.00)	0.060*		
Hospitalization in the past 3 months	2 (16.7%)	47 (9.4%)	1.92 (0.41–9.02)	0.323		

*Variables with p < 0.1 would enter the binary logistic regression analysis.

*^a^There are three missing values for this variable.*

*RO, odds ratio.*

*p < 0.05.

Subsequently, we compared the variables between individuals with and without fecal carriages of *mcr-1*-positive *E. coli* through univariate analysis. Individuals with fecal carriages of *mcr-1*-positive *E. coli* were predominantly male [Odds Ratio (OR) 8.02, 95% confidence interval (CI) 1.03–62.61] and people frequently ate chicken (OR 3.13, 95% CI 0.77–12.72), with a consumption of between 1 and 3 times per week. Another set of included individuals (OR 7.10, 95% CI 1.72–29.35) had a chicken consumption frequency of at least 3 times per week. Furthermore, antibiotic use in the past 3 months was borderline associated with *mcr-1*-positive *E. coli* carriage (OR 4.15, 95% CI 1.08–16.00) (Table 1). Through binary logistic regression analysis, chicken consumption with a higher frequency (least 3 times per week) was the only factor that had a significant association with fecal carriages of *mcr-1*-positive *E. coli* (adjust OR 6.60, 95% CI 1.58–27.62, *p* = 0.033) (Table 1).

The distribution of reasons for admission between participants with and without *mcr-1*-positive *E. coli in* fecal carriages are presented in Table 2, showing that individuals that were hospitalized due to infectious diseases had a higher proportion of *mcr-1*-positive *E. coli* in their fecal carriages than those hospitalized due to non-infectious diseases (3.6 and 0.9%, respectively, *p* = 0.041), and individuals hospitalized due to urinary tract infection (UTI) had the highest proportion (7.4%) of *mcr-1*-positive *E. coli* in their fecal carriages.

**TABLE 2:** The distribution of reasons for admitting participants with and without *mcr-1*-positive *E. coli* in their fecal carriages.

	*mcr-1* (+) *E. coli* (*n* = 12)	*mcr-1* (−) *E. coli* (*n* = 498)	*p*-value
**Admission ward**			0.119
- Baby room	0 (0)	91 (18.3%)	
- Sick baby room	3 (25.0%)	169 (33.9%)	
- Pediatric ward	9 (75.0%)	238 (47.8%)	
**Reasons for admission**			0.041*
Admission due to infectious diseases	10 (83.3%)	267 (53.6%)	
- Urinary tract infection	4	50	
- Gastroenteritis	1	57	
- Respiratory tract infections	1	79	
- Other infectious diseases	4	81	
Admission due to non-infectious diseases	2 (16.7%)	231 (46.4%)	
- Birth	0	91	
- Congenital diseases	0	33	
- Cancer	1	4	
- Other non-infectious diseases	1	103	

*p-value < 0.05.

In Table 3, we compare the antibiotic susceptibility results between *mcr-1*-positive and non-*mcr-1*-positive *E. coli* isolates determined using Vitek. Results showed that *mcr*-1-positive *E. coli* isolates were more prone to be resistant to cefoxitin (50.0% vs. 9.0%, *p* = 0.001), minocycline (41.7% vs. 15.2%, *p* = 0.031), and sulfamethoxazole-trimethoprim (75.0% vs. 40.5%, *p* = 0.018) than non-*mcr-1*-positive *E. coli* isolates. Multidrug resistance was defined as the non-susceptibility to at least 1 antibiotic in 3 or more antimicrobial categories (Magiorakos et al., 2012). Multidrug resistance (75.0% vs. 39.5%, *p* = 0.031) was also more common in *mcr-1*-positive *E. coli* isolates. Non-*mcr-1* -positive *E. coli* isolates were all susceptible to colistin. All *E. coli* isolates were susceptible to ertapenem, imipenem, amikacin, and tigecycline. Moreover, the MIC and molecular characteristics of *mcr-1*-positve *E. coli* isolates are shown in Table 4. Most *mcr-1*-positive *E. coli* isolates exhibited colistin MICs of 4–8 μg/mL. Two isolates possessed the ESBL-producing phenotype. One had *bla*_CTX–M_ and *bla*_TEM_, and the other had *bla*_TEM_. Among *mcr-1*-positive *E. coli* isolates, 10 (83.3%) belonged to ExPEC.

**TABLE 3:** Comparing antibiotic resistance profiles between *mcr-1*-positve *E. coli and* non-*mcr-1*-positve *E. coli.* using Vitek.

	*mcr-1* (+) *E. coli* (*n* = 12)	*mcr-1* (−) *E. coli* (*n* = 256)	*p*-value
Ampicillin-sulbactam	9 (75.0%)	128 (51.0%)	0.141
Piperacillin	10 (83.3%)	141 (55.1%)	0.054
Piperacillin-tazobactam	0	9 (3.5%)	1.000
Cefazolin	7 (58.3%)	100 (39.1%)	0.231
Cefoxitin	6 (50.0%)	23 (9.0%)	0.001*
Cefixime	7 (58.3%)	81 (31.6%)	0.065
Ceftriaxone	7 (58.3%)	77 (30.1%)	0.054
Ceftazidime	2 (16.7%)	38 (14.8%)	0.696
Cefepime	1 (8.3%)	21 (8.2%)	1.000
Ertapenem	0	0	
Imipenem	0	0	
Amikacin	0	0	
Gentamycin	3 (25.0%)	57 (22.4%)	0.735
Ciprofloxacin	1 (8.3%)	54 (21.1%)	0.469
Minocycline	5 (41.7%)	39 (15.2%)	0.031*
Tigecycline	0	0	
Sulfamethoxazole-trimethoprim	9 (75.0%)	102 (40.5%)	0.018*
Colistin	8 (66.7%)	0	<0.001
Multidrug resistance^a^	9 (75.0%)	100 (39.5%)	0.031*

*^a^Multidrug resistance was defined as the non-susceptibility to at least 1 antibiotic in 3 or more antimicrobial categories.*

*p-value < 0.05.

**TABLE 4:** Antibiotic susceptibility based on minimum inhibitory concentration and the molecular characteristics of 12 *mcr-1-*positive *E. coli* isolates.

Isolate no.	MIC														ESBL	ExPEC	MLST
	
	COL	SMX	TMP	CIP	TET	MERO	AZI	NAL	CTX	CHL	TGC	CFT	AMP	GEN			
No.15	4	>1024	0.5	>8	4	≤0.03	8	>16	≤0.25	16	0.5	≤0.5	8	≤0.5		+	ST-162
No.40	≤1	16	≤0.25	0.06	4	≤0.03	>64	≤4	>4	16	≤0.25	>8	>64	≤0.5	+	+	ST-108
No.91	8	>1024	>32	2	64	≤0.03	4	>128	≤0.25	>128	0.5	≤0.5	>64	1		+	ST-101
No.198	8	>1024	0.5	1	>64	≤0.03	4	>128	≤0.25	>128	≤0.25	≤0.5	8	≤0.5		+	ST-10
No.218	8	>1024	>32	0.25	>64	≤0.03	16	>128	4	128	0.25	>8	>64	1		+	ST-657
No.258	4	>1024	>32	≤0.015	64	≤0.03	>64	≤4	4	>128	≤0.25	8	>64	≤0.5			ST-58
No.290	8	>1024	>32	≤0.015	>64	≤0.03	>64	≤4	≤0.25	>128	0.5	≤0.5	>64	>32		+	ST-795
No.321	4	>1024	>32	≤0.015	64	≤0.03	4	≤4	>4	128	≤0.25	8	>64	≤0.5		+	ST-58
No.324	8	>1024	>32	≤0.015	64	≤0.03	16	≤4	4	>128	0.5	>8	>64	≤0.5		+	ST-58
No.325	4	>1024	>32	≤0.015	64	≤0.03	16	≤4	4	128	0.5	>8	>64	≤0.5		+	ST-58
No.488	4	>1024	≤0.25	≤0.015	>64	≤0.03	4	≤4	≤0.25	≤8	≤0.25	≤0.5	4	≤0.5		+	ST-847
KV-A-7-2	8	>1024	>32	0.06	>64	0.12	16	=8	>4	>128	0.5	>8	>64	>32	+	ND	ND

*COL, colistin; SMX, sulfamethoxazole; TMP, trimethoprim; CIP, ciprofloxacin; TET, tetracycline; MERO, meropenem; AZI, azithromycin; NAL, nalidixic acid; CTX, Cefotaxime; CHL, chloramphenicol; TGC, tigecycline; CFT, Ceftazidime; AMP, ampicillin; GEN, gentamicin; ESBL, extended-spectrum β-lactamase; ExPEC, extraintestinal pathogenic E. coli; MIC, minimum inhibitory concentration; MLST, multilocus sequence typing; ND, not defined.*

**FIGURE 1:**
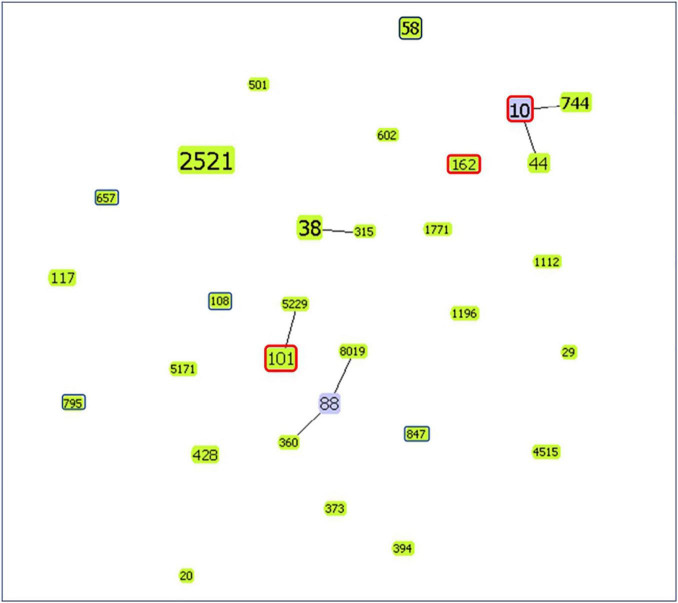
goeBURST diagram showing the genetic relatedness of *mcr-1*-positive *E. coli* isolates from the study and food animals reported in the literatures of Taiwan (Kuo et al., 2016; Liu J. Y. et al., 2020). The number within the node is the ST. Node size is based on the number of isolates with specific STs. ST of isolates in the study (from fecal samples of children) is marked with a dark blue frame. However, isolates with the same ST from the study and food animals of Taiwan reported in the literatures are marked with a red frame. Others without frame markings are ST of isolates from food animals in the literature of Taiwan. (Node color: light green: group founder; light blue: common node).

Sequence type (ST) 58 accounted for the highest proportion (*n* = 4, 33.3%). The genetic relatedness of *mcr-1*-positive *E. coli* in the study and those isolated from food animals reported in the literatures of Taiwan was evaluated using goeBURST’s algorithm (Figure 1; based on data from the MLST database)^2^ (Kuo et al., 2016; Liu J. Y. et al., 2020). Results showed that ST10, ST101, and ST162 were detected in both our participants’ fecal samples and food animals, including chicken, pork, and beef, reported in the literatures of Taiwan (Kuo et al., 2016; Liu J. Y. et al., 2020).

## Discussion

The prevalence of *mcr-1*-positive *E. coli* in fecal carriages of patients included in the study was 2.4% (12/510), which was lower than what had been reported in children (4–38.3%) from either community or hospital settings in other countries, such as China, Bolivia and India (Bi et al., 2017; Hu et al., 2017; Giani et al., 2018; Purohit et al., 2019). However, the results were similar to other recent studies in Taiwan, including data from clinical isolates and commensal *E. coli* (Lee et al., 2019; Wu et al., 2019). These findings demonstrated that the prevalence of the *mcr-1* gene was still low in Taiwan. However, an upward trend of the prevalence over time was found. Furthermore, in this study at the individual level, a significant predictor of fecal carriages in *mcr-1*-positive *E. coli* was chicken consumption. A study from China of approximately 90% adults also demonstrated that meat consumption, especially pork and mutton, was associated with *mcr-1* prevalence in a provincial region’s level (Shen et al., 2018). Another epidemiological study from southern China using vegetarian and non-vegetarian classifications did not find diet as a risk factor for *mcr-1* in fecal carriages (Wang et al., 2017b). Additionally, previous studies revealed a correlation of drug-resistance bacteria between contaminated food and the human gut. Sørensen et al. (2001) observed the same resistant strains in stool after ingestion of chickens containing those resistant bacteria. Donabedian et al. (2003) also observed related pulsed-field gel electrophoresis patterns of gentamicin-resistant isolates from human stools and meat. A recent review article indicated that foodborne transmission was a pathway of *mcr-1*-positive *E. coli* transmission (Elbediwi et al., 2019). Literature also revealed that chicken had a higher percentage of the *mcr-1* gene than other meats (Kuo et al., 2016). Therefore, chicken consumption is a possible pathway to transmit *mcr-1*-positive *E. coli* to humans. Nevertheless, more studies should investigate the detailed mechanisms.

Traveling abroad was proposed as a risk factor for drug-resistant gene transmission (Hu et al., 2020). Limited studies on the relation between traveling abroad and fecal carriages of *mcr-1*-positive *E. coli* have been reported. von Wintersdorff et al. (2016) and Nakayama et al. (2018) demonstrated that traveling to Southeast Asia or southern Africa increases the risk to *mcr-1*-positive *E. coli* in fecal carriages. However, traveling abroad was not associated with *mcr-1*-positive *E. coli* in fecal carriages in this study. The period between the time of travel and fecal collection may affect the results. In those two studies, fecal samples were collected within no more than 3 weeks after travel events. In the study presented here, travel history was traced back to 12 months before the fecal sample collection. Previous studies revealed that the proportion of drug-resistant bacterial colonization declined as time after international travels increased. For example, only about 10% of fecal colonization of ESBL *E. coli* cases remained positive after 12 months follow-up (von Wintersdorff et al., 2016; Arcilla et al., 2017; OstholmBalkhed et al., 2018). Additionally, people in Taiwan have more opportunities of contacting people from China and Southeast Asia due to their geographic location, which may weaken the influence of traveling to these countries. Therefore, more studies should investigate the relationship between international travels and *mcr-1*-positive *E. coli* in fecal carriages.

A higher proportion of participants who were hospitalized due to UTI were positive for *mcr-1*-positive *E. coli* in the study. The association between UTI pathogens and the gut microbiome had been reported before (Moreno et al., 2008; Paalanne et al., 2018). Paalanne et al. (2018) revealed that the gut microbiome was associated with the risk of febrile UTI in children. However, we did not collect urine data from the participants in the study. The relevance of UTI pathogens and fecal carriages of *mcr-1*-positive *E. coli* thus needs further study. The four participants with UTI with *mcr-1*-positive *E. coli* isolates were under 5 years. Furthermore, two of them had a history of UTI and antibiotic exposure in the last 12 months. Except for the undefined one, the other three isolates belonged to ExPEC. Interestingly, the positive rate of *mcr-1*-positive *E. coli* presence in the fecal carriage of participants with acute gastroenteritis was low (1.7%, 1/58).

Additionally, variations were observed in genotypes among the 12 *mcr-1*-positive *E. coli* cases. ST58 was the most common type in the study. *E. coli* ST58 has been isolated from different samples, including humans, the environment, food animals, and wildlife (Fuentes-Castillo et al., 2021). Furthermore, *mcr-1*-positive *E. coli* ST58 was found in chicken, turkey, cattle, and the environment (Brennan et al., 2016; Donà et al., 2017; Zurfluh et al., 2017a; Sacramento et al., 2018; Zajac et al., 2019). Similarly, *mcr-1*-positive *E. coli* ST162 was also isolated from ducks, chickens, turkeys, and dogs (Lim et al., 2016; Gelbicova et al., 2019; Zhang et al., 2019; Liu Y. Y. et al., 2020). *E. coli* ST101 and ST10 were the widespread clones that harbored *mcr-1* genes (Elbediwi et al., 2019). *mcr-1*-positive *E. coli* ST162, ST101, and ST10 have been reported in food animals and meats in Taiwan as well (Kuo et al., 2016; Liu J. Y. et al., 2020). Our preliminary data also showed that plasmid analysis from *mcr-1-*positive *E. coli* in children was similar to the plasmid sequence reported in the animals (unpublished data). Most *E. coli* ST108 have been isolated from chicken (Yang et al., 2014; Agabou et al., 2016; Pietsch et al., 2018). *E. coli* ST657 has been reported from rectal swabs of humans (Yang et al., 2014; Wang et al., 2017a). *E. coli* ST795 was found in retail vegetables and pigs (Hammerum et al., 2012; Luo et al., 2017; Xu et al., 2017). Additionally, *E. coli* ST-847 has been isolated from retail vegetables, pigs, cattle, and birds (Guenther et al., 2012; Leimbach et al., 2015; Freitag et al., 2018; Wang et al., 2020). These genotypes isolates, including ST-108, ST-657, ST-795, and ST-847, have been reported to carry different drug-resistant genes. This study is the first to reveal that these STs of *E. coli* carried the *mcr-1* gene.

Some limitations exist in this study. First, the diet information relied on self-reports provided by the participants’ parents or caregivers. Recall bias is inevitable. Second, the number of participants with *mcr-1*-positive *E. coli* in fecal carriages was relatively small, which would not detect certain risk factors. Third, *mcr-1* gene is detected mainly from *Enterobacteriaceae*. Although *E. coli* has the highest *mcr* prevalence among all bacterial species (Elbediwi et al., 2019), there are still some common bacterial species with *mcr* gene, such as *Klebsiella pneumoniae*, that have not been analyzed in the study. Furthermore, the study was limited to *mcr-1* gene, which is the most widely disseminated *mcr* gene worldwide (Ling et al., 2020). However, other *mcr* variants may exist. Last, the diversity of STs of *mcr-1*-positive *E. coli* not only means clonal expansion but also the possibility of certain plasmid transmission in the community. In our preliminary result, some of the plasmid in the *mcr-1* positive *E. coli* had the same sequence as the plasmid identified in food animals, as reported by studies published in Taiwan (unpublished data).

In conclusion, the prevalence of *mcr-1*-positive *E. coli* in fecal carriages is low among community children in Southern Taiwan who were included in this study. Nevertheless, we can found a gradual upward trend of the prevalence from 2016 to 2019. This phenomenon shows the need for continuous follow-up. A higher frequency of chicken consumption was also associated with fecal carriages of *mcr-1*-positive *E. coli*. However, the genetic relationship between our colonized isolates and isolates from chicken in Taiwan warrants further evaluation.

## Data Availability Statement

The raw data supporting the conclusions of this article will be made available by the authors, without undue reservation.

## Ethics Statement

The studies involving human participants were reviewed and approved by VGHKS 16-CT2–04, VGHKS 18-CT3–11. Written informed consent to participate in this study was provided by the participants’ legal guardian/next of kin.

## Author Contributions

P-CW, M-FC, and J-LW: conceptualization. P-CW, M-FC, W-YH, and C-HH: methodology. W-LC, M-FC, and C-HH: validation. P-CW, W-LC, and J-LW: formal analysis. P-CW and J-LW: investigation and writing—original draft preparation. W-YH and C-HH: data curation. J-LW and C-HH: writing—review and editing. C-HH: supervision. M-FC and J-LW: funding acquisition. All authors have read and agreed to the published version of the manuscript.

## Conflict of Interest

The authors declare that the research was conducted in the absence of any commercial or financial relationships that could be construed as a potential conflict of interest.

## Publisher’s Note

All claims expressed in this article are solely those of the authors and do not necessarily represent those of their affiliated organizations, or those of the publisher, the editors and the reviewers. Any product that may be evaluated in this article, or claim that may be made by its manufacturer, is not guaranteed or endorsed by the publisher.

## Footnotes

1
http://www.phyloviz.net/goeburst

2
http://mlst.warwick.ac.uk/mlst/

## Abbreviations

CI: confidence interval
*E. coli*: *Escherichia coli*
ESBL: extended-spectrum β-lactamase
ExPEC: extraintestinal pathogenic *E. coli*
MIC: minimum inhibitory concentration
MLST: multilocus sequence typing
OR: odds ratio
UTI: urinary tract infection.
